# Editorial: Gut microbiota and gastrointestinal disorders

**DOI:** 10.3389/fmed.2022.1079787

**Published:** 2022-11-03

**Authors:** Abbas Yadegar, Ali Nabavi-Rad, Javier Ochoa-Repáraz, Toshifumi Ohkusa, Yan-Dong Wang

**Affiliations:** ^1^Foodborne and Waterborne Diseases Research Center, Research Institute for Gastroenterology and Liver Diseases, Shahid Beheshti University of Medical Sciences, Tehran, Iran; ^2^Department of Biological Sciences, Boise State University, Boise, ID, United States; ^3^Department of Microbiota Research, Juntendo University Graduate School of Medicine, Tokyo, Japan; ^4^Department of Gastroenterology and Hepatology, The Jikei University Kashiwa Hospital, Chiba, Japan; ^5^State Key Laboratory of Chemical Resource Engineering, College of Life Science and Technology, Beijing University of Chemical Technology, Beijing, China

**Keywords:** gut microbiota, gastrointestinal disorders, non-alcoholic fatty liver disease, inflammatory bowel disease, fecal microbiota transplantation, short-chain fatty acid, predictive applications

Observational outcomes of recent studies indicate the notion of the gut microbiota's contribution to the host's physiological, immune, neurological, and metabolic health. In dysbiotic conditions, gut microbes could contribute to the development of a wide spectrum of diseases including metabolic syndrome, non-alcoholic fatty liver disease (NAFLD), irritable bowel syndrome (IBS), and inflammatory bowel disease (IBD) (1, 2). However, we are yet to fully understand the underlying mechanisms of the gut microbiota affecting host metabolisms and this field of study is still in its infancy; therefore, research is shifting toward cause-and-effect and multi-omics studies (3). Considering the translational applications of microbiome research, recent studies have demonstrated the great potential of gut microbiota manipulation in reducing the prevalence and severity of gastrointestinal disorders (4). Our growing knowledge about the human indigenous microbial species and the development of biotechnology and machine learning techniques are opening new avenues for the progression of microbiome-based therapeutics toward the application of precisely defined and targeted microbial consortia (5).

In this regard, the special issue of “Gut Microbiota and Gastrointestinal Disorders” appeared highly topical, wherein the aim was to provide novel insights on the central role of the gut microbiota in gastrointestinal disorders and seek innovative therapeutic and predictive applications of selected microbial species.

Considering the profound influence of the gut microbiota on the development and progression of ulcerative colitis (UC), Hu et al. reviewed the genetic, immunological, and microbial risk factors for UC pathogenesis. They enumerated potential microbiome-based approaches to restore and preserve intestinal microbial homeostasis and subsequently resolve UC. In another review, Wang et al. focused on gastrointestinal microbial dysbiosis and its contribution to the development of NAFLD, ranging from steatosis to non-alcoholic steatohepatitis (NASH). In this study, the special association of periodontopathic bacteria, mainly *Porphyromonas gingivalis*, to the pathogenesis of NAFLD was highlighted through clinical and basic research.

The significance of microbial species in the progression and emission of gastrointestinal disorders has led to the evaluation of longitudinal bio-psychological dynamics by Tavakoli et al. during the treatment procedure of IBD patients. Observational findings suggested a substantial decrease in microbial diversity of Crohn's disease (CD) patients compared to UC patients. This, in principle, leads to the interdependence of treatment strategy and microbial dynamic comparing CD and UC patients. Although most studies associating the gut microbiota with human health and disease have focused on the analysis of bacterial and archaeal components, the importance of the mycobiota, the microbiota's fungal portion, is now recognized. The analysis of the mucosa-associated mycobiota in CD patients by Olaisen et al. revealed the enrichment of *Malassezia* and the depletion of *Saccharomyces*, along with a higher proportion of *Candida albicans* and *Malassezia restricta*, compared to healthy controls. Notably, the mycobiota structural differences between the inflamed and proximal non-inflamed ileum within the same individual may contribute to CD pathogenesis. Moreover, the identification of intraindividual and interindividual differences in microbial composition can be utilized as predictive biomarkers. Tian et al. exhibited the abundance of *Alistipes* and *Eubacterium* and the impoverishment of *Roseburia* species in patients suffering from slow transit constipation. The enrichment of fatty acid biosynthesis, butanoate metabolism, and methane metabolism pathways along with microbial alteration might be potential biomarkers for slow transit constipation. Another predicting strategy was provided by Vincentis et al., evaluating the clinical improvement of patients receiving rifaximin therapy. Although inaccurate in predicting gut microbiota alterations, their electronic multi-sensorial systems constituted of e-tongue and e-nose, could effectively predict clinical improvement.

In the context of IBD research, a treatment strategy for dextran sodium sulfate (DSS)-induced colitis was evaluated by Nakajima et al. in C57BL/6 male mice. Nicotine administration prior to the induction of DSS-induced colitis elevated indole concentration in the distal colon and rectum while short-chain fatty acid (SCFA) values presented insignificant differences, compared to the control cohort. The high level of indole concentration, as well as the increased proportion of *Clostridioides* and *Porphyromonas* genera, were presented as the underpinning mechanism of DSS-induced colitis suppression following nicotine administration. Similarly, Zhou et al. reported the potential for a ginger extract to attenuate the susceptibility to DSS-induced colitis by preventing weight loss, colon shortening, inflammation, and intestinal barrier dysfunction. Ginger administration to mice models for 4 weeks following antibiotic exposure increased bacterial diversity and the relative abundance of *Helicobacter* species while decreasing the relative abundance of *Peptococcaceae* rc4-4.

Another therapeutic approach for restoring the inherent microbial composition is fecal microbiota transplantation (FMT), which was proposed by Ishikawa et al. for UC patients following triple-antibiotic therapy. This study protocol presented a double-blinded, randomized, placebo-controlled, parallel assignment trial that primarily should evaluate the Total Mayo Score at 8 weeks after the FMT procedure. Furthermore, the comparison of clinical features, microbial structure, and metabolic profile, as well as post-FMT 2-year follow-up constitute other endpoints of this study protocol.

Yang et al. assessed the efficacy and safety of FMT combined with biofeedback for mixed constipation. They reported insignificant differences in side effects, yet the combined therapy presented a substantial enrichment in the proportion of probiotic species namely *Prevotella* and *Bifidobacterium*. Considering FMT as a stopgap, yet effective and safe, microbiome-based therapeutic strategy for the remission of gut dysbiosis and gastrointestinal disorders, Savigamin et al. presented four primary steps for initiating FMT in developing countries including: 1. Finding a perfect stool donor; 2. Initiating a clinical trial; 3. Establishing a stool standard for use in other research trials; 4. Establishing a clinical center for the transplantation of fecal microbiota. They further discussed and provided insightful suggestions for the first two steps.

In conclusion, the studies published in this Research Topic further shed light on the critical contribution of the gut microbiota in the pathogenesis of gastrointestinal disorders and its potential capacity in developing efficient biomarkers and therapeutic strategies. In this field of study, the knowledge gaps to be filled and the bottlenecks to be surpassed include deciphering the contribution of hundreds of as-yet-unknown metabolites and their related signaling pathways to the host's physiological and metabolic health and disease.

## Author contributions

AN-R wrote the first draft of the manuscript. AY, JO-R, TO, and Y-DW revised the manuscript. All authors read and approved the submitted version.

## Conflict of interest

The authors declare that the research was conducted in the absence of any commercial or financial relationships that could be construed as a potential conflict of interest.

## Publisher's note

All claims expressed in this article are solely those of the authors and do not necessarily represent those of their affiliated organizations, or those of the publisher, the editors and the reviewers. Any product that may be evaluated in this article, or claim that may be made by its manufacturer, is not guaranteed or endorsed by the publisher.
